# Attachment Styles and Parenting Attitudes in Adults With “Pseudo-Attention-Deficit/Hyperactivity Disorder”

**DOI:** 10.7759/cureus.70362

**Published:** 2024-09-28

**Authors:** Yuki Sawamura, Hitomi Hirokawa-Ueda, Reiko Taketani, Hisae Ono

**Affiliations:** 1 Department of Psychological Science, Graduate School of Humanities, Kwansei Gakuin University, Nishinomiya-shi, JPN; 2 Department of Psychology, Faculty of Human Sciences, Konan Women’s University, Kobe-shi, JPN

**Keywords:** adult attention deficit/hyperactivity disorder, attachment style, fear of abandonment, parental attitudes, pseudo-attention deficit/hyperactivity disorder

## Abstract

Background

The number of individuals who experience the onset of attention-deficit/hyperactivity disorder (ADHD)-like symptoms after the age of 12 years has been growing, which does not meet the diagnostic criteria for adult ADHD. We designated this condition as “pseudo-ADHD” and investigated its psychological implications by comparing the parenting attitudes in childhood and attachment styles among individuals with “pseudo-ADHD,” those with adult ADHD, and healthy controls.

Methods

We conducted an online cross-sectional survey of adults aged 18 years. Participants in the analysis included individuals with “pseudo-ADHD” (n = 46), adults with ADHD (n = 23), and healthy controls (n = 758). The Parental Bonding Instrument (PBI) was used to evaluate parenting attitudes, and the Experiences in Close Relationships Inventory for the Generalized Other (ECR-GO) was used to evaluate attachment styles. One-way analysis of variance was performed to compare the PBI and ECR-GO scores among the three groups.

Results

Regarding the PBI scores, a significant main effect of group was found for the paternal care scores (p = 0.002), and the “pseudo-ADHD” group did not differ significantly from the adult ADHD group or healthy control group (p = 0.378, p = 0.228, respectively). A significant main effect of the group was found for the maternal care scores (p < 0.001). The “pseudo-ADHD” group scored significantly lower than the adult ADHD group (p = 0.005), whereas there was no significant difference compared to the healthy control group (p = 1.000). A significant main effect of the group was also found for the paternal overprotection scores (p < 0.001). The “pseudo-ADHD” group scored significantly lower than the adult ADHD group (p = 0.002), whereas there was no significant difference compared to the healthy control group (p = 0.571). A significant main effect of group was found for the maternal overprotection scores (p < 0.001), and the “pseudo-ADHD” group did not differ significantly from either the adult ADHD group or the healthy control group (p = 0.060, p = 0.161, respectively). Regarding ECR-GO scores, a significant main effect of group was found for the fear of abandonment score (p < 0.001), and the “pseudo-ADHD” group scored significantly lower than the adult ADHD group (p < 0.001) and significantly higher than the healthy control group (p = 0.005).

Conclusion

This study suggested that the parenting attitudes of parents of individuals with “pseudo-ADHD” were not different from those of the healthy controls. For attachment styles, the fear of abandonment was higher in individuals with “pseudo-ADHD” than in healthy controls, but not as high as in patients with adult ADHD. This study highlights, for the first time, the importance of considering attachment styles in the diagnosis and treatment of individuals with ADHD symptoms in adulthood.

## Introduction

Recently, the number of adults suspected of having attention-deficit/hyperactivity disorder (ADHD) seeking medical care has been increasing in Japan [1,2]. However, among these individuals, some strongly complain of the onset of ADHD-like symptoms such as difficulty concentrating and frequently forgetting things or making mistakes after the age of 12 years [2], which do not meet the diagnostic criteria for adult ADHD as outlined in the Diagnostic and Statistical Manual of Mental Disorders, Fifth Edition, Text Revision (DSM-5-TR) [3]. The diagnostic criteria for adult ADHD according to the DSM-5-TR include (A) a persistent pattern of inattention and/or hyperactivity-impulsivity that interferes with functioning or development (for adults, at least five symptoms are required for each of inattention and/or hyperactivity-impulsivity), (B) several inattentive or hyperactive-impulsive symptoms were present prior to age 12 years, (C) several inattentive or hyperactive-impulsive symptoms are present in two or more settings, (D) there is clear evidence that the symptoms interfere with, or reduce the quality of, social, academic, or occupational functioning, and (E) the symptoms do not occur exclusively during schizophrenia or another psychotic disorder and are not better explained by another mental disorder [3]. This may be because these individuals represent a distinct group with a different pathology from that of adults with ADHD [4]; however, no empirical research has been conducted on the psychological pathology of this condition. Therefore, we designated this condition as “pseudo-ADHD” and aimed to explore its psychological implications.

Inappropriate parenting and unstable attachment styles during childhood have been reported as factors that increase the likelihood of adult ADHD symptoms, with or without a diagnosis of adult ADHD [5,6]. The inclusion of individuals regardless of their diagnostic status means that it encompasses not only adults with diagnosed ADHD but also those with “pseudo-ADHD.” Adult ADHD is predominantly influenced by innate biological factors, as suggested by a genome-wide meta-analysis that identified significant risk variants at 12 independent loci [7]. However, "pseudo-ADHD", which is characterized by the absence of symptoms during childhood, is believed to have fewer biological underpinnings than adult ADHD. Thus, psychosocial factors, such as parenting attitudes and unstable attachment styles, may play a more prominent role as contributing factors to “pseudo-ADHD.”

Therefore, the purpose of this study was to compare differences in parenting attitudes in childhood and attachment styles in adulthood among three groups: individuals with “pseudo-ADHD,” those with adult ADHD, and healthy controls. We hypothesized that individuals with “pseudo-ADHD” were most affected by these psychosocial factors. This study would provide a better psychological understanding of “pseudo-ADHD,” facilitate differential diagnosis with adult ADHD, and provide direction for psychosocial treatment of “pseudo-ADHD.”

## Materials and methods

Study design

The present study was a cross-sectional online survey conducted in February 2023. This online survey was conducted through a crowdsourcing service (Lancers: https://www.lancers.jp/). The sampling method was non-probability sampling. The data analyzed in this study were obtained from the “Study of Related Factors in Symptoms of Adult Attention-Deficit/Hyperactivity Disorder” (UMIN ID: 000050196).

Participants

The inclusion criteria for all participants were (1) individuals who understand the purpose of the research and can provide written consent to participate, (2) individuals who are 18 years old or older at the time of study participation, and (3) individuals who completed all the response items. We did not define exclusion criteria for all participants. From those participants, the following three groups were selected and analyzed: the “pseudo-ADHD” group, including individuals who had not been diagnosed with ADHD, presented ADHD symptoms in adulthood, and did not present ADHD symptoms in childhood; the adult ADHD group, including individuals who had been diagnosed with ADHD and presented with ADHD symptoms in adulthood and in childhood; and the healthy control group, including individuals who had not been diagnosed with ADHD and did not present ADHD symptoms in adulthood and childhood.

Individuals who had been diagnosed with ADHD but did not have ADHD symptoms in adulthood, as well as those who had ADHD symptoms in childhood but no longer have ADHD symptoms in adulthood, were excluded from the study. Additionally, individuals who were diagnosed with ADHD despite not having ADHD symptoms during childhood, as well as those who have experienced ADHD symptoms in both childhood and adulthood but have not been diagnosed, were also excluded from this study.

Survey procedures

Informed consent was obtained from all the participants online. The survey was designed and distributed using an online platform (Qualtrics). An honorarium was awarded to each participant. This study was approved by the Institutional Review Board for Medical and Biological Research Involving Human Subjects of Kwansei Gakuin University (approval number: KG-IRB-22-06).

Measures

A self-administered questionnaire was used to assess participants' characteristics. Participants were instructed to respond to three questions regarding sex: male, female, and no response. Occupations based on the Japanese occupational classification [8] were categorized as employed, unemployed, student, or other. Additionally, participants were instructed to respond with “yes” or “no” on whether or not they have been diagnosed with ADHD, based on their self-reports, including diagnoses made during both childhood and adulthood.

The Adult ADHD Self-Report Scale (ASRS) was used to assess ADHD symptoms [9,10]. This scale uses a five-point scale (never, rarely, sometimes, often, and very often) to rate how often the participants observed behaviors and feelings related to ADHD symptoms in the past six months. The ASRS consists of 18 questions on ADHD symptoms in adulthood, and each question is divided into Parts A and B. Part A consists of six questions. If four or more of the six questions in Part A were answered, the respondent was considered to have ADHD symptoms during adulthood. In this study, ADHD symptoms in adulthood were identified when four or more of the six questions in Part A were answered. We downloaded and used this scale from a website where it was freely available under an open copyright [10].

The Wender-Utah Rating Scale (WURS) was used to assess childhood ADHD symptoms [11,12]. It consists of 25 items assessing childhood ADHD symptoms. Respondents recalled their childhood behaviors and assessed how often they recognized behaviors associated with ADHD symptoms on a five-point scale (not at all or very slightly, mildly, moderately, quite a bit, or very much). If the WURS total score was 46 or higher, childhood ADHD symptoms were likely to be present. In this study, ADHD symptoms in childhood were identified to be present when the WURS total score was 46 or higher. We obtained permission from the author who has a copyright of the scale to use this scale in the study by referring to the paper [11].

The Parental Bonding Instrument (PBI) was used to assess parenting attitudes during childhood. It consists of 25 questions on paternal and maternal parenting attitudes [13,14]. Respondents recalled their paternal and maternal parenting attitudes up to age 16 and rated them on a four-point scale (very unlike, moderately unlike, moderately like, and very like). PBI consists of two factors: the care factor and the overprotection factor, and the paternal and maternal care scores and overprotection scores were calculated. Care scores ranged from 0 to 36, and overprotection scores ranged from 0 to 39. Higher scores indicated stronger attitudes toward parental care or overprotection. We obtained permission from the author who has a copyright of the scale to use this scale in the study by referring to the paper [13].

The Experiences in Close Relationships Inventory for the Generalized Other (ECR-GO) was used to assess attachment styles in adulthood [15]. ECR-GO consists of 20 items on adult attachment styles for the generalized other. The feelings and sensations commonly experienced in interpersonal relationships were rated on a seven-point scale (strongly disagree, disagree, slightly disagree, neutral, slightly agree, agree, and strongly agree). ECR-GO consists of two factors: fear of abandonment and avoidance of intimacy. The scores range from 10 to 70, with higher scores indicating a greater fear of abandonment or avoidance of intimacy. We obtained permission from the author who has a copyright of the scale to use this scale in the study by referring to the paper [15].

Outcome

The primary outcome was the difference in scores on the four subscales of the PBI (maternal care, paternal care, maternal overprotection, and paternal overprotection) and the two subscales of the ECR-GO (fear of abandonment and avoidance of intimacy) among the “pseudo-ADHD,” adult ADHD, and healthy control groups.

Statistical analysis

A one-way analysis of variance was performed to examine the differences in scores on the subscales of the PBI and ECR-GO. The Bonferroni correction was used for multiple comparisons. IBM SPSS Statistics 29 (IBM Corp., Armonk, NY, USA) was used for the statistical analysis. The chi-square test and ANOVA were used to analyze the data, and statistical significance was set at p < 0.05.

## Results

Characteristics of the participants

The number of participants was 1,017. Table 1 presents the characteristics of the “pseudo-ADHD” group, the adult ADHD group and the healthy control group. The “pseudo-ADHD” group consisted of 46 participants (4.52%), the adult ADHD group consisted of 23 (2.26%), and the healthy control group consisted of 758 (74.53%). Although they were not included in the analysis of this study, there were 72 individuals (7.08%) who had ADHD symptoms in both childhood and adulthood but had not been diagnosed. Age was significantly lower in the “pseudo-ADHD” and adult ADHD groups than in the healthy controls (p = 0.029, p < 0.001, respectively). The ratio of participants who responded with “student” was significantly lower in the “pseudo-ADHD” and healthy control groups than in the adult ADHD group. The number of participants who responded with “other” as their occupation was significantly higher in the “pseudo-ADHD” and adult ADHD groups than in the healthy control group.

**Table 1:** Characteristics of participants in the three groups. ADHD: Attention-Deficit/Hyperactivity Disorder, SD: standard deviation Values in parentheses indicate percentages within the group. Sex and employment were analyzed using the χ^2^ test and age using one-way analysis of variance. The value of χ^2^ for sex was 5.33 and that for employment was 14.23.

	Pseudo-ADHD group (N = 46)	Adult ADHD group (N = 23)	Healthy control group (N = 758)	P-value
Sex				0.256
Male (%)	29 (63.04)	12 (52.17)	361 (47.63)	
Female (%)	16 (34.78)	11 (47.83)	389 (51.32)	
No-response (%)	1 (2.17)	0 (0.00)	8 (1.06)	
Age±SD	38.89±10.74	33.87±9.82	42.94±10.27	< 0.001
Employment				0.027
Employed (%)	30 (65.22)	13 (56.52)	493 (65.04)	
Unemployed (%)	6 (13.04)	4 (17.39)	175 (23.09)	
Student (%)	1 (2.17)	2 (8.70)	10 (1.32)	
Other (%)	9 (19.57)	4 (17.39)	80 (10.55)	

Comparison of PBI and ECR-GO subscale scores among the groups

**Figure 1:**
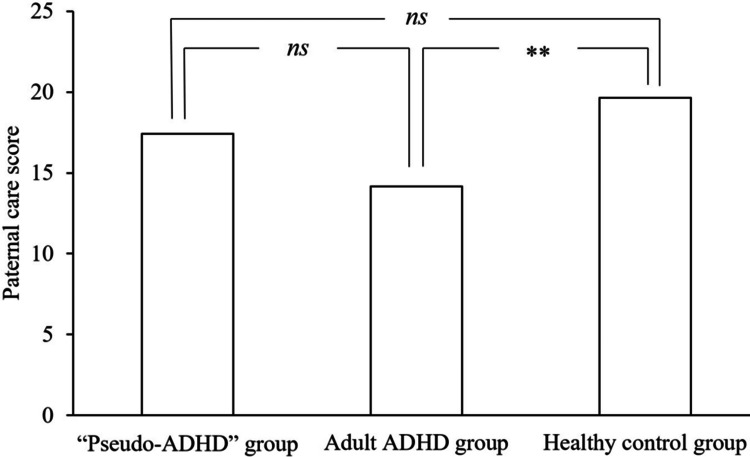
Comparison of paternal care of the PBI in the three groups. ADHD: Attention-Deficit/Hyperactivity Disorder, PBI: Parental Bonding Instrument, ns: not significant The mean score±standard deviation for paternal care was 17.41±9.19 in the “pseudo-ADHD” group, 14.17±7.90 in the adult ADHD group, and 19.65±8.24 in the healthy control group. The F-value of the one-way analysis of variance was 6.235. **p<0.01

**Figure 2:**
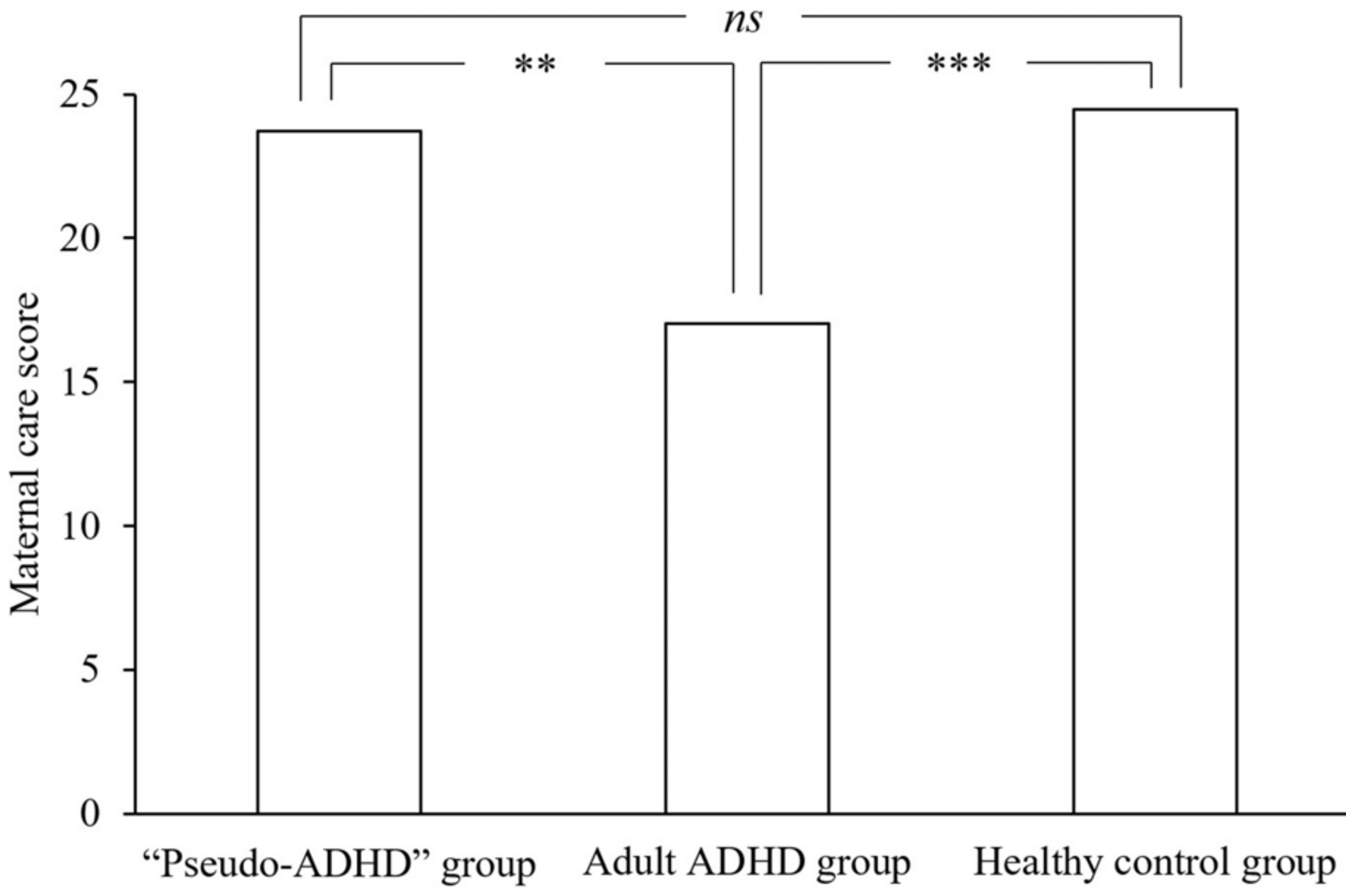
Comparison of maternal care of the PBI in the three groups. ADHD: Attention-Deficit/Hyperactivity Disorder, PBI: Parental Bonding Instrument, ns: not significant The mean score±standard deviation for maternal care was 23.72±8.05 in the “pseudo-ADHD” group, 17.04±7.91 in the adult ADHD group, and 24.47±8.38 in the healthy control group. The F-value of the one-way analysis of variance was 8.926. ***p<0.001, **p<0.01

**Figure 3:**
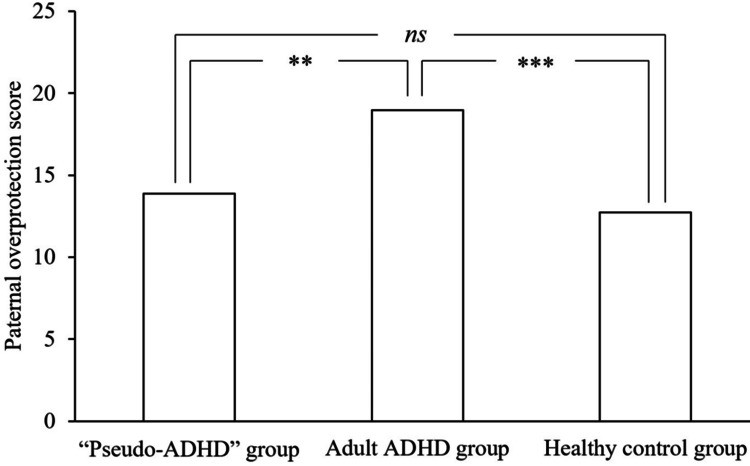
Comparison of paternal overprotection of the PBI in the three groups. ADHD: Attention-Deficit/Hyperactivity Disorder, PBI: Parental Bonding Instrument, ns: not significant The mean score±standard deviation for paternal overprotection was 13.87±6.01 in the “pseudo-ADHD” group, 18.96±6.75 in the adult ADHD group, and 12.73±5.67 in the healthy control group. The F-value of the one-way analysis of variance was 13.829. ***p<0.001, **p<0.01

**Figure 4:**
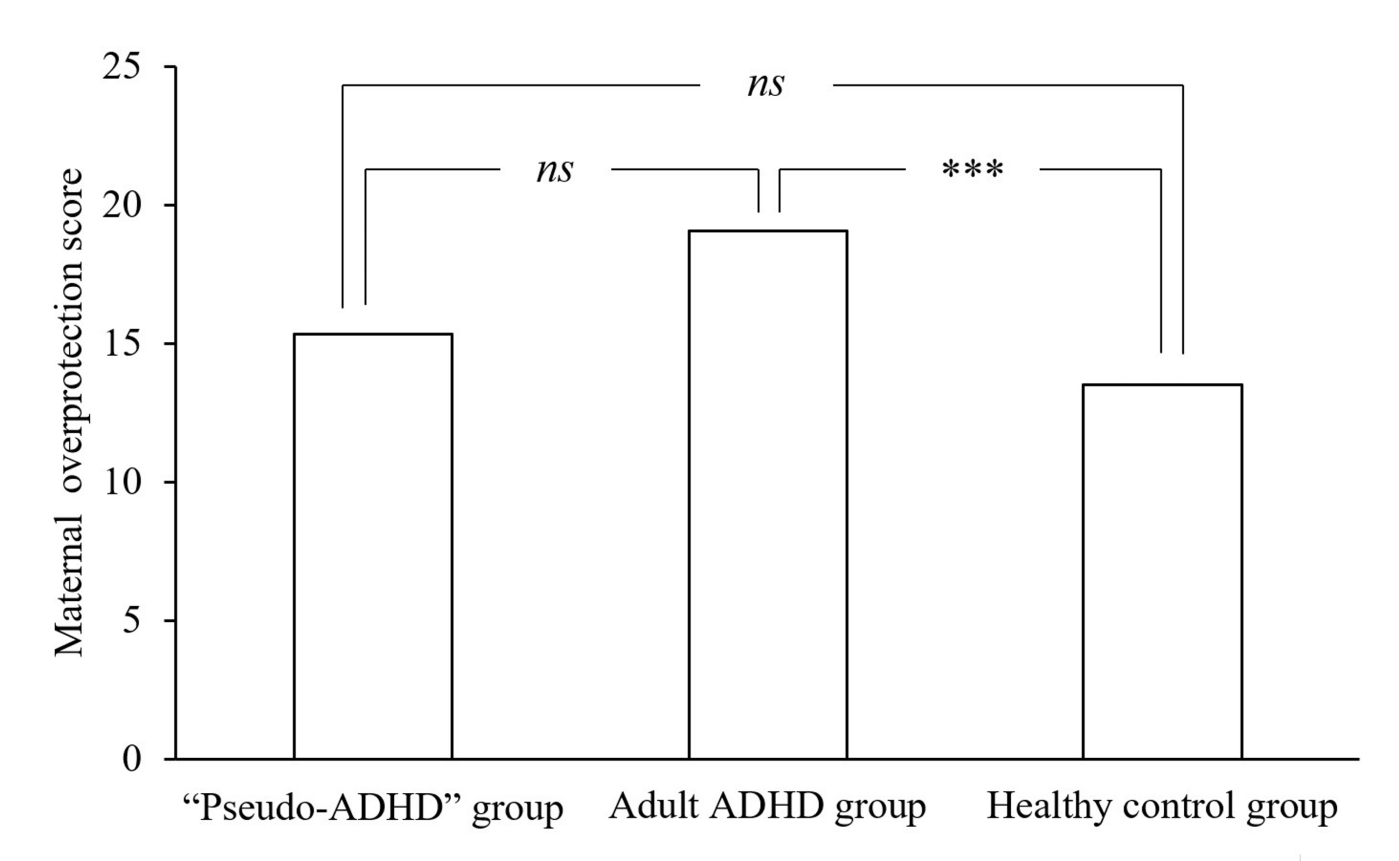
Comparison of maternal overprotection of the PBI in the three groups. ADHD: Attention Deficit/Hyperactivity Disorder, PBI: Parental Bonding Instrument, ns: not significant The mean score±standard deviation for maternal overprotection was 15.35±6.50 in the “pseudo-ADHD” group, 19.09±7.65 in the adult ADHD group, and 13.51±6.22 in the healthy control group. The F-value of the one-way analysis of variance was 10.371. ***p<0.001

Table 2 shows the subscale scores of PBI and ECR-GO in the “pseudo-ADHD”, adult ADHD, and healthy control groups. Figures 1-4 show the PBI subscale scores in the three groups. A significant main effect of group was found for the paternal care scores (F (2, 824) = 6.235, p = 0.002), and the “pseudo-ADHD” group did not differ significantly from either group. However, the adult ADHD group scored significantly lower than the healthy control group (p = 0.006). A significant main effect of group was found for the maternal care scores (F (2, 824) = 8.926, p < 0.001), and the “pseudo-ADHD” group scored significantly higher scores than the adult ADHD group (p = 0.005), but not significantly different from the healthy control group. In contrast, the adult ADHD group scored significantly lower than the healthy control group (p < 0.001). A significant main effect of group was found for the paternal overprotection score (F (2, 824) = 13.829, p < 0.001), and the “pseudo-ADHD” group scored significantly lower than the adult ADHD group (p = 0.002), but not significantly different from the healthy control group. The adult ADHD group had significantly higher scores than the healthy control group (p < 0.001). A significant main effect of group was found for the maternal overprotection score (F (2, 824) = 10.371, p < 0.001), but the scores of the “pseudo-ADHD” group did not differ significantly from those of either group. However, the adult ADHD group showed significantly higher scores than the healthy control group (p < 0.001).

**Table 2:** Subscale scores of PBI and ECR-GO in the three groups. ADHD: Attention-Deficit/Hyperactivity Disorder, PBI: Parental Bonding Instrument, ECR-GO: The Experiences in Close Relationships inventory for the Generalized Other PBI and ECR-GO subscale scores in the three groups were indicated as mean±standard deviation. The Bonferroni correction was used for multiple comparisons of PBI and ECR-GO subscale scores in the three groups.

	"Pseudo-ADHD" group (N = 46)	Adult ADHD group (N = 23)	Healthy control group (N = 758)	P-value of one-way analysis of variance	P-value of "Pseudo-ADHD" group vs healthy control group	P-value of "Pseudo-ADHD" group vs adult ADHD group	P-value of adult ADHD group vs healthy control group
PBI							
Paternal care score	17.41±9.19	14.17±7.90	19.65±8.24	0.002	0.228	0.378	0.006
Maternal care score	23.72±8.05	17.04±7.91	24.47±8.38	< 0.001	1.000	0.005	< 0.001
Paternal overprotection score	13.87±6.01	18.96±6.75	12.73±5.67	< 0.001	0.571	0.002	< 0.001
Maternal overprotection score	15.35±6.50	19.09±7.65	13.51±6.22	< 0.001	0.161	0.060	< 0.001
ECR-GO							
Fear of abandonment score	35.43±10.03	46.09±12.50	30.51±10.27	< 0.001	0.005	< 0.001	< 0.001
Avoidance of intimacy score	45.87±9.22	45.96±14.64	45.01±10.83	0.809	1.000	1.000	1.000

**Figure 5:**
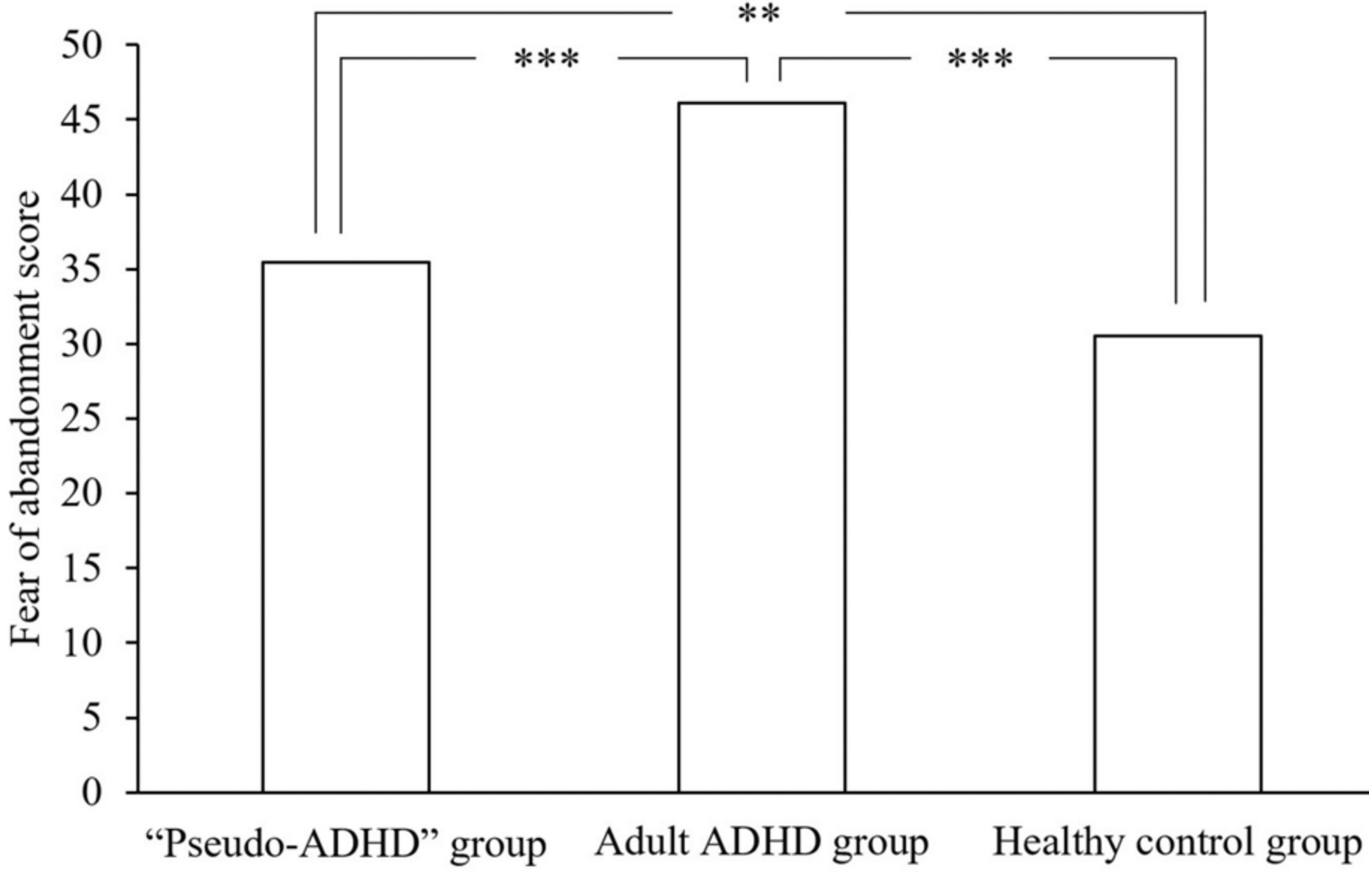
Comparison of fear of abandonment of the ECR-GO in the three groups. ADHD: Attention-Deficit/Hyperactivity Disorder, ECR-GO: The Experiences in Close Relationships inventory for the Generalized Other The mean score±standard deviation for fear of abandonment was 35.43±10.03 in the “pseudo-ADHD” group, 46.09±12.50 in the adult ADHD group, and 30.51±10.27 in the healthy control group. The F-value of the one-way analysis of variance was 29.481. ***p<0.001, **p<0.01

**Figure 6:**
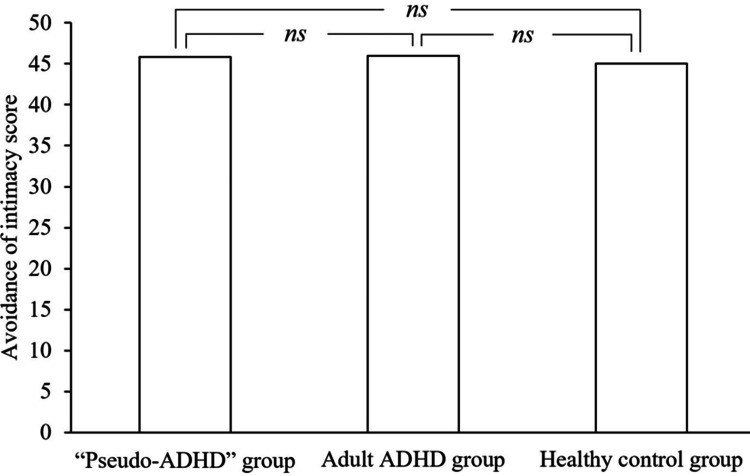
Comparison of avoidance of intimacy of the ECR-GO in the three groups. ADHD: Attention-Deficit/Hyperactivity Disorder, ECR-GO: The Experiences in Close Relationships inventory for the Generalized Other, ns: not significant The mean score±standard deviation for avoidance of intimacy was 45.87±9.22 in the “pseudo-ADHD” group, 45.96±14.64 in the adult ADHD group, and 45.01±10.83 in the healthy control group. The F-value of the one-way analysis of variance was 0.211.

Figures 5, 6 show the ECR-GO subscale scores for the three groups. A significant main effect of group was found for the fear of abandonment score (F (2, 824) = 29.481, p < 0.001), with the “pseudo-ADHD” group scoring significantly lower than the adult ADHD group (p < 0.001) and significantly higher than the healthy control group (p = 0.005). Additionally, the adult ADHD group showed significantly higher scores than the healthy control group (p < 0.001). However, no main effect of group was observed for the intimacy avoidance score, indicating no significant difference among the three groups (F (2, 824) = 0.211, p = 0.809).

## Discussion

This study was the first to examine the parenting attitudes and adult attachment styles of individuals with “pseudo-ADHD” compared to those with adult ADHD and healthy controls. The results showed that the parenting attitudes of individuals with “pseudo-ADHD” were not different from those of healthy controls. For attachment styles, it was revealed that fear of abandonment was higher in individuals with “pseudo-ADHD” than in healthy controls, but not as high as in patients with adult ADHD. Furthermore, avoidance of intimacy was not significantly different among the groups. Therefore, our hypothesis that “pseudo-ADHD” is more affected by parenting attitudes and attachment styles than adult ADHD was not supported.

In this study, 1,017 participants were included, of whom approximately 5% had “pseudo-ADHD” and 2% had adult ADHD. The percentage of patients with adult ADHD is 2%-3% [16], which is consistent with the results of this study. Thus, the proportion of “pseudo-ADHD” identified in this study may be generalizable. Given that “pseudo-ADHD” appears to occur at twice the rate of adult ADHD, a thorough assessment of childhood ADHD symptoms is crucial to prevent potential overdiagnosis. Conversely, approximately 3% of adults who have not been diagnosed with ADHD were reported to exhibit ADHD symptoms in adulthood [17], underscoring the issue of underdiagnosis. This finding highlights the importance of confirming the presence of ADHD symptoms during childhood when assessing patients presenting with ADHD symptoms at medical institutions. Furthermore, we consider it necessary to understand the pathology of those who did not have ADHD symptoms during childhood and address their treatment.

This study showed that the parenting attitudes of parents of individuals with “pseudo-ADHD” did not differ from those of parents of healthy individuals. However, this study also demonstrated that the parenting attitudes of parents of patients with adult ADHD were not favorable. In addition to the inherent difficulties in raising a child with ADHD, approximately 17% of parents of patients with ADHD have ADHD [18]. Parents’ characteristics may further destabilize parenting attitudes.

The present study also showed that the severity of fear of abandonment in attachment style in “pseudo-ADHD” was intermediate between that of healthy controls and adult ADHD patients. Although it has been reported that the attachment style of adult ADHD patients is unstable [19], it was possible that “pseudo-ADHD” may also have an unstable attachment style, although not to the same extent as adult ADHD patients.

We considered the possibility that interpersonal distortions resulting from fear of abandonment may be underlying the ADHD-like symptoms showed by “pseudo-ADHD.” Recently, in Japan, there has been an environment in which information about ADHD is readily available through television and the Internet [20], and reasonable accommodation in the workplace and educational settings for adult patients with ADHD is expanding [21,22]. Therefore, individuals with a fear of abandonment may misattribute their social adaptation difficulties to adult ADHD and seek a diagnosis. This suggested the necessity of thoroughly assessing ADHD symptoms, including an evaluation of fear of abandonment, to inform effective treatment strategies.

The main limitations of this study were as follows. First, this study used data from a cross-sectional survey to compare groups, and causal relationships were not examined. Second, the participants were asked to recall and self-report their parenting attitudes during childhood and ADHD diagnoses; thus, there was respondent bias. Furthermore, it is unclear whether ADHD diagnoses were made during childhood or adulthood. Third, this study was likely conducted with a large population interested in ADHD. Participant selection bias may have occurred, because this study was an online survey. Fourth, the number of participants in the adult ADHD group was fewer than 30, resulting in unequal sample sizes among the three groups.

## Conclusions

This study examined parenting attitudes and attachment styles among those with “pseudo-ADHD,” defined as those who had not been diagnosed with ADHD, presented ADHD symptoms in adulthood, and did not present ADHD symptoms in childhood. The results showed no difference in parenting attitudes of parents of individuals with “pseudo-ADHD” compared to those of healthy controls. However, the results indicated that the severity of fear of abandonment in individuals with “pseudo-ADHD” was intermediate between that of healthy controls and adults with ADHD. Individuals with a fear of abandonment may attribute their difficulties in social adaptation to adult ADHD and seek a diagnosis. Further studies that conduct group comparisons with equal sample sizes and incorporate objective assessments of ADHD symptoms, along with standardized diagnostic criteria, are necessary.
